# Water pre-filtration methods to improve environmental DNA detection by real-time PCR and metabarcoding

**DOI:** 10.1371/journal.pone.0250162

**Published:** 2021-05-07

**Authors:** Kazuto Takasaki, Hiroki Aihara, Takanobu Imanaka, Takahiro Matsudaira, Keita Tsukahara, Atsuko Usui, Sora Osaki, Hideyuki Doi

**Affiliations:** 1 Research and Development Division, FASMAC Co., Ltd., Atsugi, Kanagawa, Japan; 2 Biotechnological Research Support Division, FASMAC Co., Ltd., Atsugi, Kanagawa, Japan; 3 Graduate School of Information Science, University of Hyogo, Kobe, Hyogo, Japan; University of Waikato, NEW ZEALAND

## Abstract

Environmental DNA (eDNA) analysis is a novel approach for biomonitoring and has been mostly used in clear water. It is difficult to detect eDNA in turbid water as filter clogging occurs, and environmental samples contain various substances that inhibit the polymerase chain reaction (PCR) and affect the accuracy of eDNA analysis. Therefore, we applied a pre-filtration method to better detect the fish species (particularly pale chub, *Opsariichthys platypus*) present in a water body by measuring eDNA in environmental samples containing PCR inhibitors. Upon conducting 12S rRNA metabarcoding analysis (MiFish), we found that pre-filtration did not affect the number or identities of fish species detected in our samples, but pre-filtration through pore sizes resulted in significantly reduced variance among replicate samples. Additionally, PCR amplification was improved by the pre-filtration of environmental samples containing PCR inhibitors such as humic substances. Although this study may appear to be a conservative and ancillary experiment, pre-filtration is a simple technique that can not only improve the physical properties of water, such as turbidity, but also the quality of eDNA biomonitoring.

## Introduction

Environmental DNA (eDNA) analysis is a novel approach to investigating the species distribution for environmental monitoring and conservation [1–3], which allows species to be detected without observation or direct capture. Thus, this approach is an environmentally friendly and cost-effective tool for early monitoring systems [4–6]. eDNA methods were developed for detecting specific species based on the real-time polymerase chain reaction (PCR) [1] and, more recently, quantitative PCR (qPCR) [2, 3], and they are still widely used in species distribution analysis. Based on high-throughput sequencing (HTS), eDNA metabarcoding has since been widely used as a method of rapid biodiversity assessment [7–9]. However, both eDNA approaches require optimisation to increase the likelihood of detection, particularly when analysing water with varying eDNA conditions [10, 11].

There are multiple methods of capturing (concentrating), purifying (extracting), and amplifying eDNA [12–14]. Most eDNA studies that use a filtration approach have been conducted in marine or freshwater systems, where water appears to be non-turbid at the time of collection [15–18]. This is owing to the unique set of challenges that turbid water poses when detecting eDNA, such as the clogging of filters and the presence of PCR inhibitors [19–21]. Previous studies have utilised extraction kits—which come with anti-inhibitory washes—various pore sizes, membrane types, and pre-filtration steps to prevent filter clogging [22–25]. In this study, we focused on the effect of pre-filtration techniques on removal of inhibitors and effective detection of target organisms’ eDNA.

Humic substances, such as humic and fulvic acid, are common in aquatic, soil, and sedimentary environments [26], and play important roles in freshwater treatment by interacting with toxic heavy metals and trihalomethanes [27]. On the other hand, a previous study reported that trace amounts of humic substances can inhibit the PCR and cause false-negative results [28–30]. Therefore, to achieve optimal eDNA analysis results, humic substances must be removed from the studied water samples. However, whether pre-filtration affects the presence of PCR inhibitors in water samples is unclear.

Therefore, in this study, we investigated whether pre-filtration removed humic acid, a PCR inhibitor, and improved the species detection of the eDNA approach following two methods: 1) eDNA metabarcoding with amplicon sequencing to investigate the impact of pre-filtration on the detection of fish communities, and 2) species-specific eDNA detection by qPCR to investigate the impact of pre-filtration on the detection of a fish species and evaluate the inhibition of the PCR.

## Methods

### Study site

The site considered in this study is the Sagami River, which is the largest river in Kanagawa Prefecture, Japan, and a popular place for recreational activities, such as fishing. To protect and sustain these recreational activities, Kanagawa Prefecture continuously monitors the aquatic communities of the Sagami River, and particularly the fish communities. As the fish communities of the Sagami River are well-studied, it is a suitable site for validating the results of this study. The Sagami River is also the closest water body to our laboratory, which minimised the time required for transporting water samples between the river and the lab. Therefore, we analysed water samples collected from the Sagami River in the tests conducted in this work.

### Environmental water sampling

**Fig 1:**
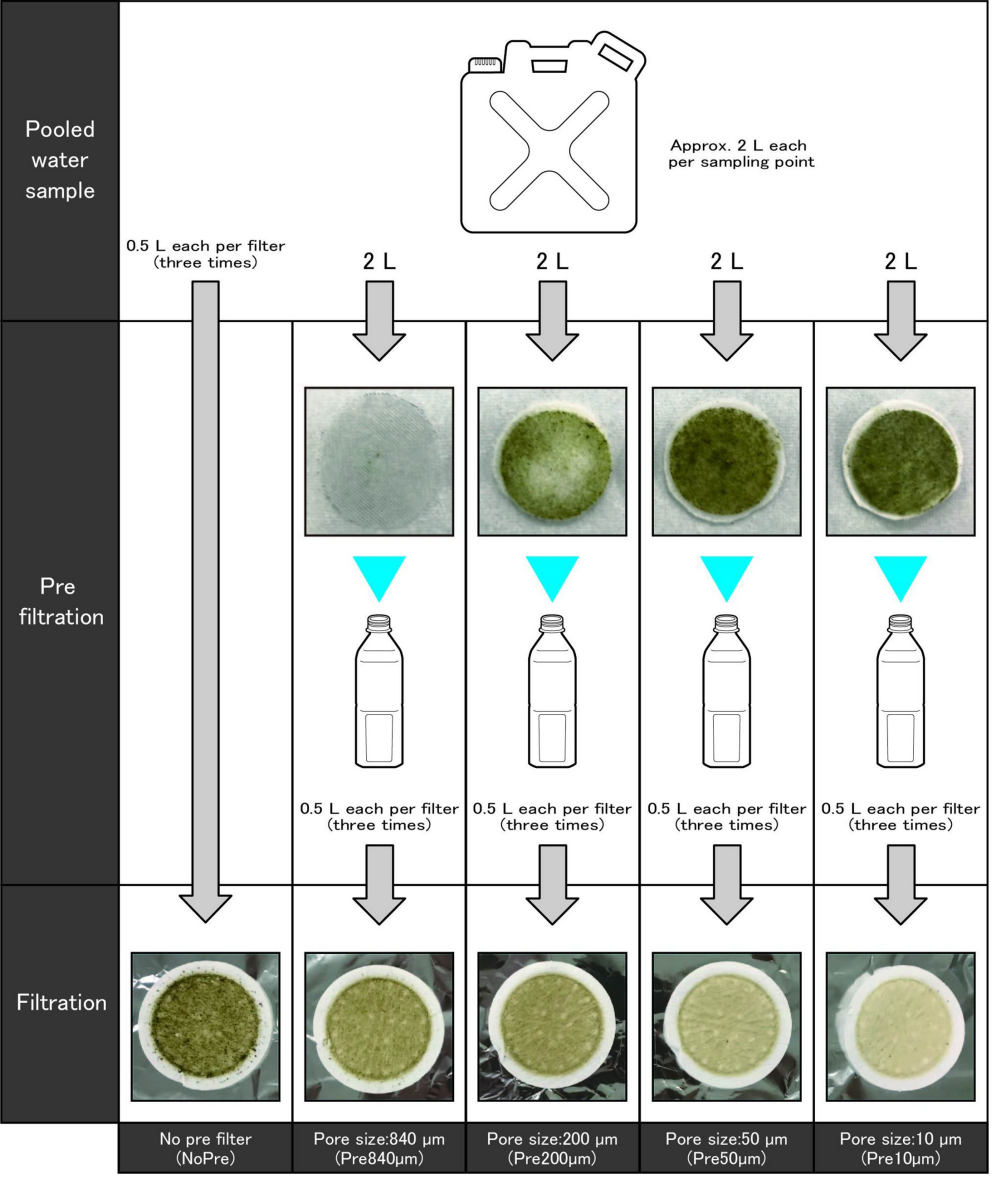
Overview of the pre-filtration technique used in the experiment.

Environmental water samples were collected from the Sagami River system in Kanagawa Prefecture, Japan [latitude: 35.318725–35.58872222; longitude: 139.2714528–139.3789833], using disposable plastic bottles and immediately pooled into a plastic tank on site (Fig 1) [31]. Water samples were collected independently on four sampling dates: June 9, 2018 (MiSeq sequencing); September 16, 2018 (IPC-targeted qPCR assay, and species-specific qPCR assay); December 22, 2018 (MiSeq sequencing); and February 3, 2020 (IPC-targeted qPCR with coffee filter). Additionally, 2 mL of Osban S (Takeda Pharmachemical Co. Ltd., Japan) containing *10 w/*v% benzalkonium chloride was added to the tank to preserve the eDNA [32] in the pooled water. The pooled water was immediately transported to the laboratory for filtration. All equipment was cleaned using 0.6% hypochlorous acid and washed with DNA-free distilled water. No permits were required for the collection and analysis of the samples.

### Pre-filtration of environmental water

We prepared four types of polypropylene filters with pore sizes of 840, 200, 50, and 10 μm for pre-filtration. The filters with a pore size of 840 μm were purchased from Dio Chemicals, Ltd. (Tokyo, Japan), and the other three were purchased from 3M Japan Ltd. (Tokyo, Japan). The pre-filters were cut into circles with a diameter of 47 mm and then placed in a disposable filter funnel (Nihon Pall Ltd., Tokyo, Japan) after removing the originally attached funnel membrane. The prepared pre-filters were set on a portable in-line pump (Nihon Pall Ltd., Japan), and 2 L of the pooled water was passed through each filter (Fig 1). The pre-filtered water samples were then divided into 500 mL subsamples, with three samples per treatment (e.g., four pre-filtration pore sizes and no pre-filtration), and filtered through a 47 mm glass microfiber filter, Grade GF/F (normal pore size of 0.7 μm; Whatman, Maidstone, UK), as shown in Fig 1. Each filter was wrapped in aluminium foil and stored at −20°C before DNA extraction.

### eDNA extraction

The eDNA was extracted from each filter using a DNeasy Blood and Tissue Kit (Qiagen, Hilden, Germany) and a commercial spin column following the protocol reported by Miya *et al*. [7]. The extracted DNA was then purified using the DNeasy Blood and Tissue Kit following the manufacturer’s protocol.

### Paired-end library preparation and MiSeq sequencing

A two-step tailed PCR approach was followed for library preparation using paired-end sequencing on the MiSeq platform (Illumina, CA, U.S.A). Prior to library preparation, the workspace and equipment were sterilised, and filtered pipette tips were used. One PCR blank was included per set of reactions during library preparation to monitor contamination. In the first PCR, a target region of the mitochondrial 12S rRNA gene was amplified using the MiFish-U-F and MiFish-U-R primers (forward: 5′-*ACACTCTTTCCCTACACGACGCTCTTCCGATCT*NNNNNNGTCGGTAAAACTCGTGCCAGC-3′; reverse: 5′-*GTGACTGGAGTTCAGACGTGTGCTCTTCCGATCT*NNNNNNCATAGTGGGGTATCTAATCCCAGTTTG-3′). The italicised and non-italicised letters represent the MiSeq sequencing primers and MiFish-U primers, respectively. Additionally, six random bases (N) were used to enhance cluster separation in the MiSeq flow cells during the initial base-call calibrations on the MiSeq platform. The experiment was conducted with a reaction volume of 13 μL, including 6.0 μL of 2 × KAPA HiFi Hot Start ReadyMix (KAPA Biosystems, Wilmington, MA), 0.7 μL of each primer (5 μm), 2.6 μL of sterile distilled water, and 2.0 μL of the extracted DNA as a template. The thermal cycle profile was as follows: initial denaturation at 95°C for 3 min, followed by 28 cycles of denaturation at 98°C for 20 s, annealing at 65°C for 15 s, and elongation at 72°C for 15 s, followed by final elongation at the same temperature for 5 min. The first PCR was replicated eight times per sample, and the eight replicated samples were either not pooled (the environmental water collected on June 9, 2018) or pooled (the environmental water collected on December 22, 2018) and purified using an Agencourt AMPure XP kit (Beckman Coulter, CA, U.S.A.). Though the first PCR replicates are usually pooled in the MiFish metabarcoding method, some of the samples in this study were not pooled in order to monitor the increase in species in relation to an increase in replicates. The purified first PCR products were used as templates for the second PCR, which was amplified using primers containing a dual-indexed sequence (octoX) to identify each sample and adapter sequences bound to the flow cell (forward: 5′-*AATGATACGGCGACCACCGAGATCTACA*XXXXXXXXACACTCTTTCCCTACACGACGCTCTTCCGATCT-3′; reverse: 5′-*CAAGCAGAAGACGGCATACGAGAT*XXXXXXXXGTGACTGGAGTTCAGACGTGTGCTCTTCCGATCT-3′). The italicised and non-italicised letters represent the MiSeq P5/P7 adapter and sequencing primers, respectively. The eight "X"s in the bases represent the dual-index sequences inserted to identify the different samples. The second PCR was conducted with a 13 μL reaction volume containing 6.0 μL of 2 × KAPA HiFi HotStart ReadyMix, 0.7 μL of each primer (5 μm), 3.6 μL of sterile distilled water, and 1.0 μL of the template. The thermal cycle profile was as follows: initial denaturation at 95°C for 3 min, followed by eight cycles of denaturation at 98°C for 20 s, annealing and elongation at 72°C for 15 s, and final elongation at the same temperature for 5 min. Each product was purified using an Agencourt AMPure XP kit. After quantifying the DNA using a **Qubit fluorometer with** a Qubit dsDNA HS assay kit (Thermo Fisher Scientific, MA, U.S.A) **and** Agilent BioAnalyzer with high-sensitivity DNA chips (Agilent Technologies, CA, U.S.A), the PCR products were pooled in equimolar proportions, and their volumes were adjusted following the protocol for Illumina MiSeq platform sequencing. A 30% Phix spike-in control was added to the pooled library to improve data quality. Sequencing was conducted using the MiSeq platform with V2 reagent to generate 2 × 250-bp paired-end reads. We conducted the aforementioned post-PCR steps, including PCR and MiSeq sequencing, in separate DNA extraction and water filtration rooms to avoid DNA contamination.

### MiSeq sequencing data analysis

Fastq files (raw reads) of each sample were generated by demultiplexing using MiSeq Reporter software version 1.3.17.0 (Illumina, CA, U.S.A). Data pre-processing and raw read analyses were conducted with the following scripts and steps: (1) primer sequences were removed from both the forward and reverse reads using fastx_barcode_splitter.pl and fastx_trimmer in the FASTX Toolkit 0.0.14 (available from http://hannonlab.cshl.edu/fastx_toolkit); (2) to merge the paired reads, the pre-processed reads were analysed using R version 3.5.1 and DADA2 library version 1.8.0 (available from http://benjjneb.github.io/dada2/), following DADA2 Pipeline Tutorial 1.12 (https://benjjneb.github.io/dada2/tutorial.html); (3) the pre-processed reads from the aforementioned pipeline were dereplicated using the ’unique’ command of R, and the number of identical reads was added to the header line of the FASTA formatted data file (a table of the number of detected reads in each sample to unique sequences was created from this data); (4) the processed reads were then subjected to local BLASTN searches against MitoFish, and the top BLAST hit with an E-value threshold of 6^−77^ was applied for assigning the species of each representative (unique) sequence.

### IPC-targeted qPCR assay

To estimate the PCR sensitivity in the presence of PCR inhibitors under each pre-filtration condition, we conducted qPCR after adding humic substances (Canadian humin HNC, purchased from PIC-BIO, Inc., Tokyo, Japan) to the environmental water sample from the Sagami river system (collected on September 16, 2018) [29, 33, 34]. We prepared water with a humin content of 1 g L^−1^ for robust PCR inhibition according to a previous study [29]. However, as filtration under some conditions failed owing to filter clogging, the concentration was set to 250 mg L^−1^ to enable filtration under all conditions. Humin-free water samples were prepared for each pre-filtration condition to act as a control. The pre-filtered water samples were then divided into 500 mL subsamples (repeated twice as biological replicates) and filtered through a GF/F filter with a diameter of 47 mm.

We also preliminary tested the suitability of using coffee filters to filter humic water. We tested three different commercial coffee filter setups (purchased from Toyo Trading Co., LTD. (Aichi, Japan). The environmental water collected from the Sagami River system on February 3, 2020 was divided into samples of 500 mL and filtered (the positive control was replicated twice, and the pre-filtration conditions were replicated thrice as biological replicates). The humic substance was added to the environmental water samples at a concentration of 250 mg L^−1^ for pre-filtration, and the water filtration process and controls were the same as those in the previous experiment.

An Internal Positive Control (IPC, 20 copies μL^-1^; Nippon Gene, Toyama, Japan) was used to assess the PCR sensitivity. Twenty copies of the IPC were added to 5 μL of DNA extracted from each filtered sample. The IPC8-5′and IPC8-3′primers (forward: 5′-CCGAGCTTACAAGGCAGGTT-3′; reverse: 5′-TGGCTCGTACACCAGCATACTAG-3′) were used for amplification, and the IPC was detected using an IPC1-Taq: 5′-(FAM) TAG CTT CAA GCA TCT GGC TGT CGG C (TAMRA)-3’ hydrolysis probe. The qPCR assay was conducted using a Thermal Cycler Dice ® Real-Time System Lite (Takara, Shiga, Japan), and three PCR replicates were prepared as technical replicates. A reaction volume of 20 μL consisted of 10 μL of the THUNDERBIRD® qPCR Mix (Toyobo, Osaka, Japan), 0.6 μL of each primer (10 μM), 0.4 μL of the probe, 2.4 μL of sterile distilled water, 1.0 μL IPC (20 copies), and 5 μL of extracted DNA. The thermal cycle profile was as follows: initial denaturation at 95°C for 30 s, followed by 45 cycles of denaturation at 95°C for 10 s, and annealing and elongation at 60°C for 30 s. The obtained data were analysed using Thermal Cycler Dice ® Real-Time System Lite Software ver. 5.00 (Takara, Shiga, Japan). In parallel, we tested nuclease free water for negative control, and no negative controls were detected (data not shown).

### Species-specific qPCR assay

To estimate the PCR specificity in the presence of PCR inhibitors under each pre-filtration condition, we added 30 mg L^−1^ of humic substances to the environmental water samples to recreate natural conditions [35, 36]. Humin-free water samples were prepared as controls. Two-litre water samples were filtered through each pre-filter. The pre-filtered water was then divided into 500 mL subsamples (repeated three times as biological replicates) and filtered through a GF/F filter with a diameter of 47 mm.

Pale chub (*O*. *platypus*) was targeted in this study to estimate the species specificity. The qPCR primers, probe, and thermal cycle conditions were consistent with those reported by Kitanishi *et al*. [37] (S3 Table). THUNDERBIRD® qPCR Mix or TaqMan Environmental Master Mix 2.0 (Thermo Fisher Scientific, MA, U.S.A) were used as the qPCR master mix in these experiments. Three PCRs were prepared as technical replicates. The qPCR and data analysis were conducted using the same equipment and process as those of the previous experiment. In parallel, we tested water for negative control, and none were detected (data not shown).

### Statistical analysis

All statistical analyses were conducted in R ver. 3.6.0 [38], and all significance levels were set to α = 0.05. We conducted NMDS to visualise the dissimilarity of the communities based on incidence-based Jaccard and abundance-based Bray-Curtis indices. The NMDS scores and stress were calculated with 999 separate runs of real data. We evaluated the differences between the community structures of the pre-filtration conditions by conducting permutational multivariate analysis of variance (PERMANOVA) with the Jaccard similarity matrix and 999 permutations. We used the "metaMDS" and "adonis" functions of "vegan" ver. 2.5.6 for NMDS and PERMANOVA, respectively.

We tested the differences in the threshold cycle (Ct) values of the IPC and pale chub detection between the different pre-filter sizes and the presence/absence of humin by conducting a two-way repeated-measure ANOVA with the interaction using the "aov" function. We conducted a Tukey post-hoc test for the pre-filter conditions with the presence/absence of humin separately using the "TukeyHSD" function because of the significant interaction (see Results). For the coffee filter experiment and number of fish species, we tested the differences between the pre-filter sizes by conducting a repeated-measure ANOVA using the "aov" function and performed a Tukey post-hoc test as previously described. For all ANOVA tests, we preliminary tested the normality of the data by performing the Shapiro-Wilk test using the R function “shapiro.test” and verified the normality of all datasets (W > 0.846, p < 0.0001).

## Results

### Reproducibility of fish communities under each pre-filtration condition

**Fig 2:**
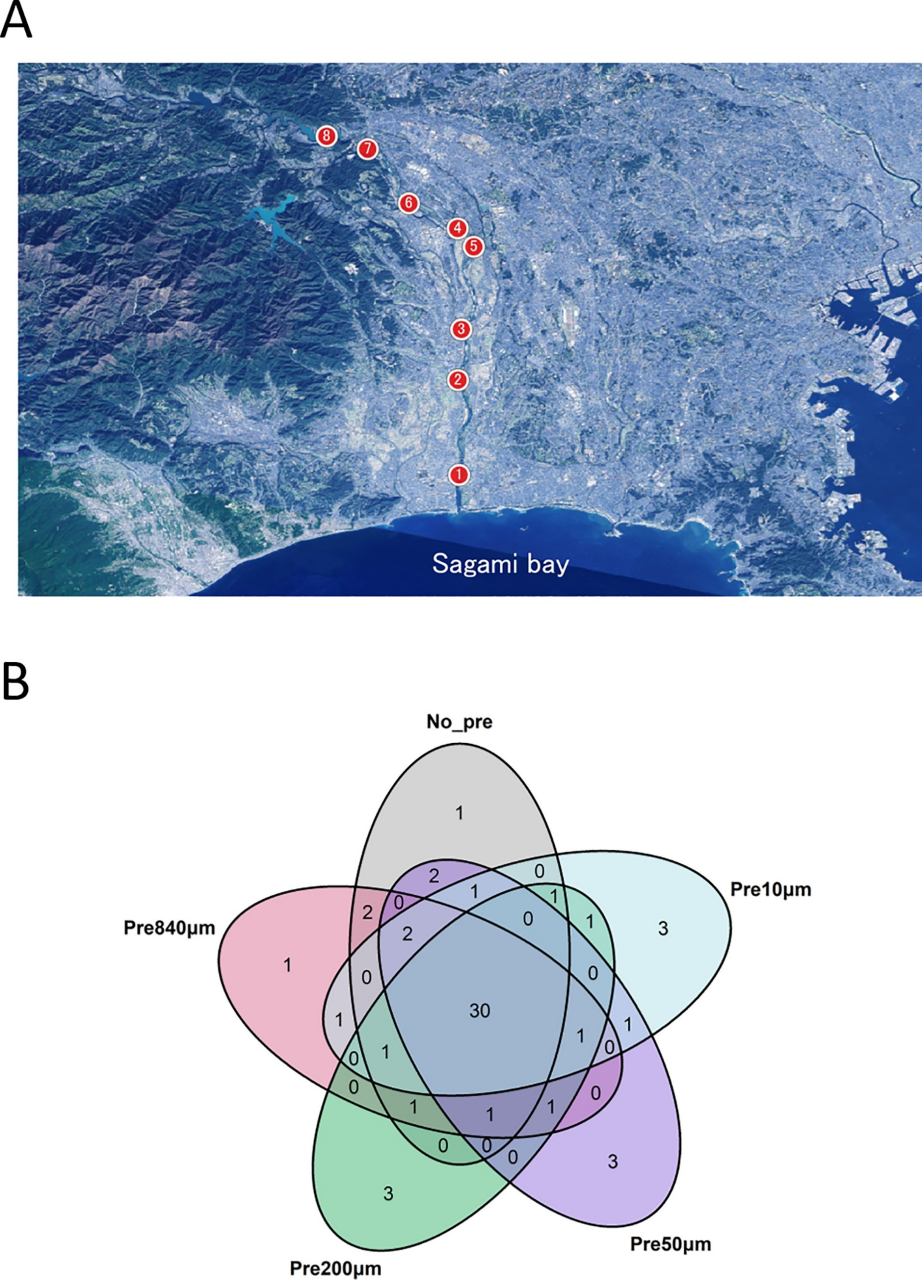
Reproducibility of the fish communities by 12S rRNA amplicon analysis (MiFish) under each pre-filtration condition. (A) Map showing the sampling points in closed circles on 9 June, 2018. This map was created using QGIS version 2.14 based on the map tile in the Geospatial Information Authority of Japan (https://maps.gsi.go.jp/development/ichiran.html). [Data source of the map tiles] Landsat8 image (GSI, TSIC and GEO Grid/AIST), Landsat8 image (courtesy of the U.S. Geological Survey), Submarine topography (GEBCO). (B) Venn diagram showing the number of shared species between each pre-filtration condition.

We evaluated the reproducibility of fish communities by 12S rRNA amplicon analysis (MiFish) of the water samples obtained from the eight sites on June 9, 2018, using MiSeq. We identified 57 species in 120 PCR amplifications (no pre-filtration [NoPre] condition, and four pre-filtration conditions; 840 μm [Pre840μm], 200 μm [Pre200μm], 50 μm [Pre50μm], and 10 μm [Pre10μm], with three filtration replicates and eight PCR replicates) from the eDNA samples (S1 Table). Additionally, the number of detected species increased when increasing the number of PCR replicates under all conditions (S1 Fig). The total numbers of species detected from the three water sample replicates were not significantly different between the pre-filtration conditions (ANOVA, F = 0.123, p = 0.971; Fig 2B; NoPre: 42 spp., Pre840μm: 41 spp., Pre200μm: 40 spp., Pre50μm: 42 spp., and Pre10μm: 42 spp., total species).

We evaluated the reproducibility of fish communities for each pre-filtration condition using a non-metric multidimensional scaling (NMDS) ordination by incidence-based Jaccard indices (S3A Fig; NMDS stress = 0.21) and abundance-based Bray-Curtis indices (S3B Fig; NMDS stress = 0.032), and observed significant differences between the fish communities identified when testing different pre-filtration pore sizes (PERMANOVA, F = 2.34, p = 0.03), indicating that the variety of the fish communities converged as the pre-filtration pore sizes reduced. We also employed the NMDS by abundance-based Bray-Curtis index, and achieved similar results (S4B Fig, PERMANOVA, F = 2.34, p = 0.03). Furthermore, most of the 30 species detected under all conditions were included in the major group of fish communities (S1 Table). The same test was conducted using water samples collected on December 22, 2018, and similar tendencies were observed (S4A Fig; NMDS stress = 0.192, S4B Fig; NMDS stress = 0.067 and S2 Table, PERMANOVA, F = 2.49, p = 0.017).

### PCR sensitivity in the presence of inhibitors under each pre-filtration condition

**Fig 3:**
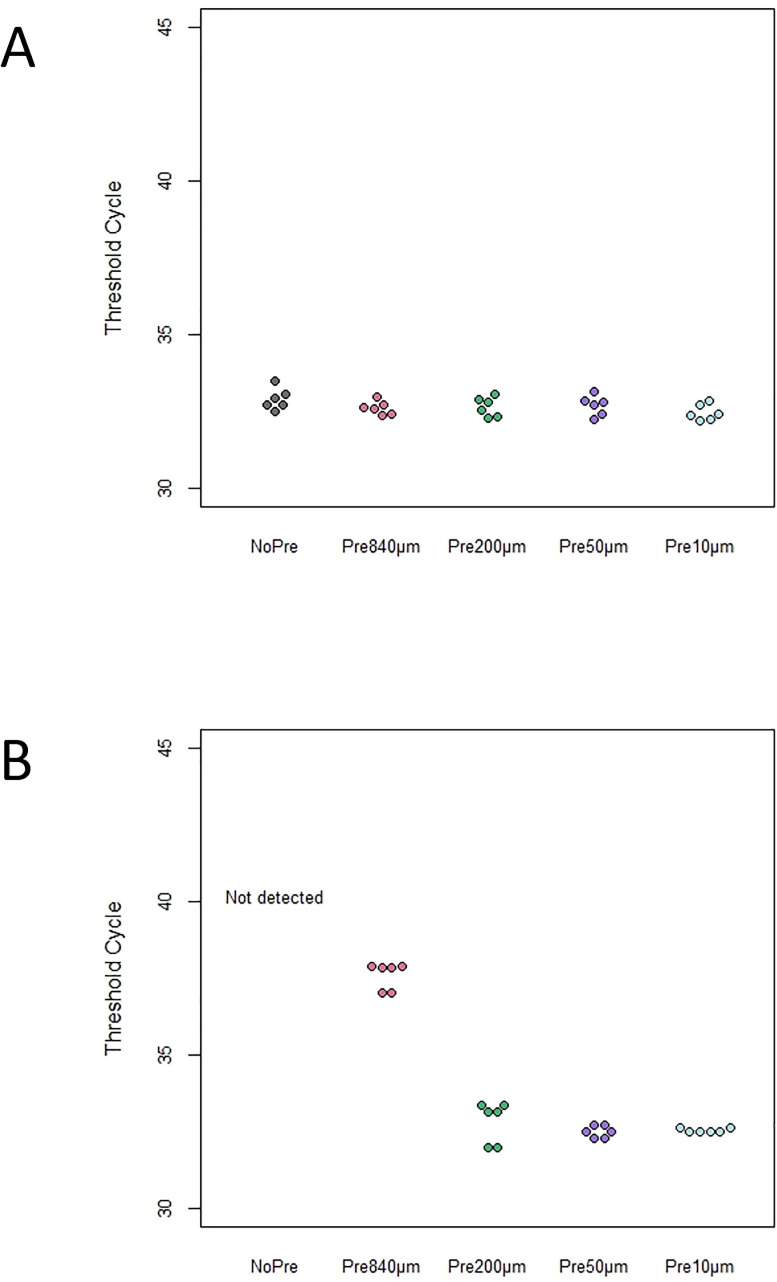
IPC detection assay (Threshold cycle, Ct) under each pre-filtration condition by qPCR. (A) In the absence of humin; (B) in the presence of humin (IPC amplification failed for all replicates on NoPre and are denoted as “Not detected”).

We tested whether PCR sensitivity in samples containing humin was improved by pre-filtration (Fig 3). In the presence of humin, the IPC amplifications failed for all replicates of the NoPre condition (Fig 3B). However, for each pre-filtration condition, the amplifications of all replicates were successful. The threshold cycle (Ct) values significantly differed depending on the pre-filtration pore sizes, presence of humin, and the interaction (two-way ANOVA, F = 113.3, 150.4, 146.9, respectively, p < 0.001 for all), indicating that pre-filtration reduced PCR inhibition due to the presence of humin. Additionally, the differences between the PCR of the samples pre-filtrated with different pore sizes in the absence of humin were not significant (Tukey, p > 0.125), while the difference between the 840 μm-filtered samples and those filtered through the other sizes was (200, 50, and 10 μm, Tukey, p < 0.0001).

**Fig 4:**
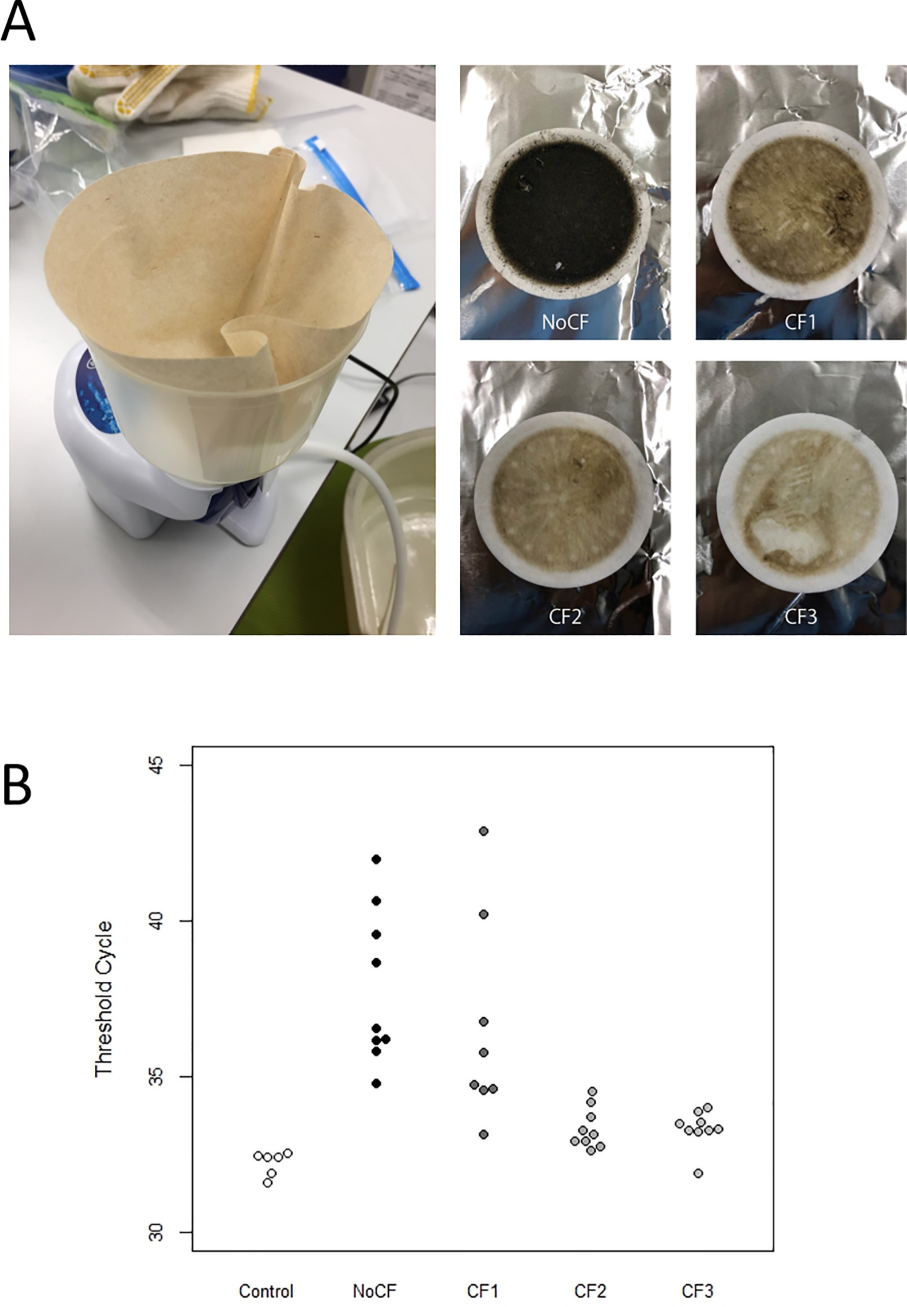
Tests for the three conditions in the presence of humin using commercial coffee filters for pre-filtration. (A) Model of the coffee and GF/F filters after filtration under each condition. (B) DNA detection assay (Threshold cycle, Ct) results for pale chub for each coffee pre-filtration by qPCR. Control, NoCF, CF1, CF2, and CF3 refer to the conditions with humin-free water samples, no coffee filter, and single, double, and triple coffee filters for humin-containing water samples, respectively.

We also conducted tests for the three conditions in the presence of humin using commercial coffee filters (Fig 4A) for pre-filtration with a lower cost than that incurred when using membrane filters. We observed significant differences in the Ct depending on the number of coffee filters [ANOVA, F = 12.6 (p < 0.0001)], indicating that the use of coffee filters could improve species-specific detection. Furthermore, we observed significantly greater inhibitor effects on No_pre compared with both CF2 and CF3, as well as on CF1 compared with both CF2 and CF3 (Tukey, p < 0.0143, Fig 4B).

### Species-specific analysis under each pre-filtration condition

**Fig 5:**
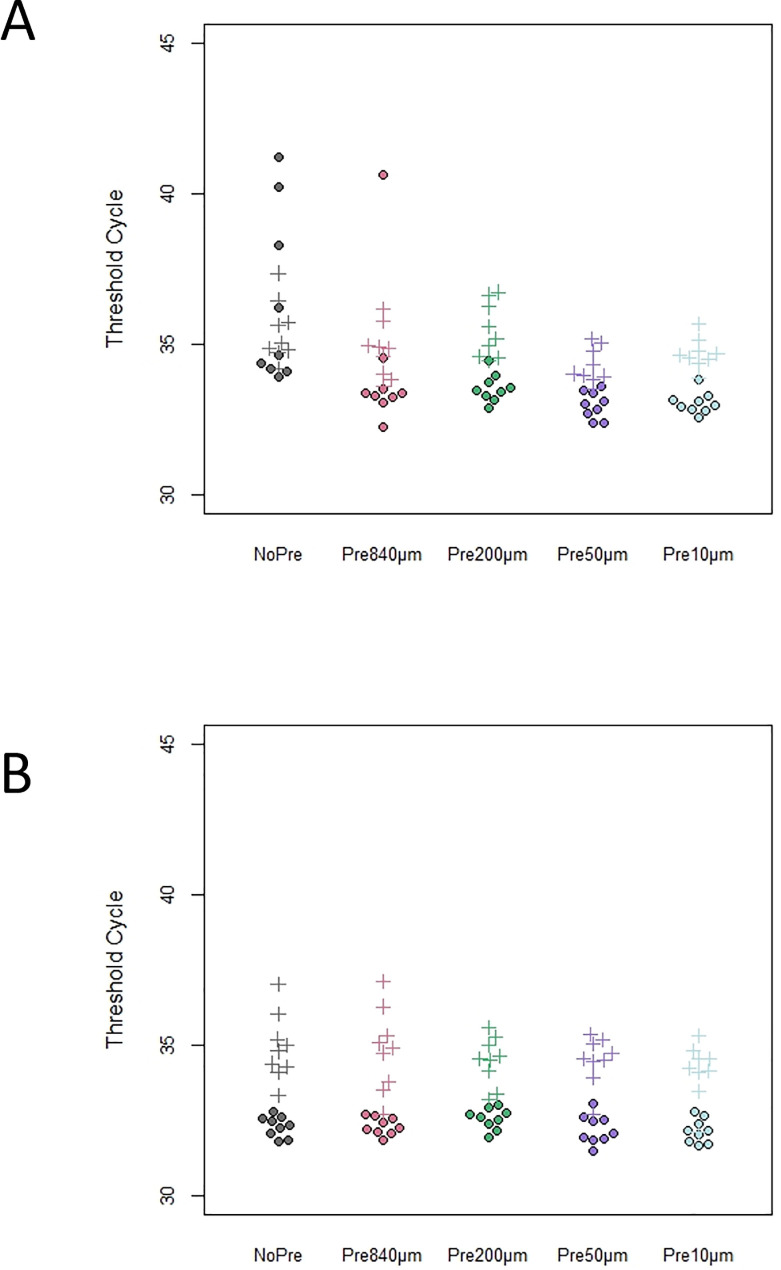
DNA detection assay (Threshold cycle, Ct) results for pale chub under each pre-filtration condition by qPCR. (A) In the absence of humin; B) in the presence of humin. Closed circles and daggers indicate the results detected by the THUNDERBIRD® qPCR Mix and Environmental Master Mix 2.0, respectively.

We considered that pre-filtration may prevent species-specific detection through the premature removal of the eDNA source, as eDNA source collection failed when using filters with pore sizes larger than 1 μm in a previous study [39]. To confirm the species specificity of each pre-filtration condition, we conducted a pale chub (*Opsariichthys platypus*)-specific qPCR assay, as it is the most common fish in the Sagami River system, following a previously reported qPCR-based genotyping method [40]. The amplifications were successful under all conditions (Fig 5A and 5B). Furthermore, the Ct values were significantly different depending on the pre-filtration pore sizes, presence of humin, and the interaction [two-way ANOVA, F = 4.28 (p = 0.0033), F = 40.2 (p < 0.0001), and F = 2.58 (p = 0.0423), respectively], indicating that pre-filtration improved species-specific detection. Furthermore, there was a non-significant difference in the Ct values of the samples pre-filtered with different pore sizes in the absence of humin (Tukey, p > 0.305), and a significant difference between No_pre and the other sizes (840, 50, and 10 μm, Tukey, p < 0.028), excluding 200 μm (p = 0.224). In eDNA studies employing qPCR, the Environmental Master Mix 2.0 (Thermo Fisher Scientific, MA, U.S.A) is widely used for its tolerance to PCR inhibitors; therefore, we also conducted qPCR using this reagent and eDNA sample (Fig 5A and 5B). There were non-significant differences between the Ct values of the samples pre-filtered with different pore sizes and in the presence/absence of humin [two-way ANOVA, F = 2.48 (p = 0.051), F = 3.33 (p = 0.072), and F = 1.28 (p = 0.286), respectively], indicating that the reactivity was stable in both the presence and absence of humin.

## Discussion

In this study, we investigated whether pre-filtration affects the analysis of fish communities using eDNA methods and whether it can effectively remove PCR-inhibiting substances. By conducting experiments using samples from a natural river system, we confirmed that pre-filtration significantly affected the detection of species by qPCR using species-specific primers, and by metabarcoding using MiFish universal amplification. In the qPCR conducted using the species-specific primer, the performance of the < 200 μm mesh size was significantly better than that of the No_pre and 840 μm mesh size. In MiFish metabarcoding, the fish community converged as the pre-filter pore size decreased. Therefore, we demonstrated that pre-filtration increased the performance of eDNA surveys in the presence of inhibitory humic compounds, and pre-filtration with small pore-sizes (e.g., 200 μm) appeared to improve performance.

Using eDNA metabarcoding with pre-filters, we detected common Japanese fish species, such as *Tribolodon hakonensis*, *Zacco platypus*, and *Nipponocypris temminckii*, irrespective of the pre-filter pore size (S1 Table). Additionally, the data also confirmed the presence of *Hemibarbusbarbus sp*. and *Rhynchocypris lagowskii*, which are endemic to Japan, and *Squalidus* sp., which are rare freshwater fish species in the Kanagawa area [40]. Hence, the results indicate that pre-filtration did not affect the detection of the species richness and community compositions. Invasive alien species (IAS) are a major threat to the biodiversity of native species [41, 42]. *Micropterus salmoides* and *Lepomis macrochirus* are two major IAS in Japan and popular recreational river-fishing targets and were also detected in this work. Therefore, our eDNA analysis approach involving pre-filtration can be applied in conservation and monitoring, as previously reported [22]. Following pre-filtration, the selection of appropriate filters, including pore size and filter material, may also significantly impact eDNA retention [43].

Pre-filtration is expected to increase the amount of water filtered and eDNA yield by preventing filter clogging by turbid water. Current water preparation methods have lower eDNA yields per filter owing to filter clogging, particularly in wetlands and turbid ponds [44]. Our study site has turbid water and is one of the 500 important wetlands in Japan, designated by the Ministry of the Environment, Japan, as it is inhabited by rare species (http://www.env.go.jp/nature/important_wetland/index.html, in Japanese, Accessed on 3 June 2020). It is important to apply eDNA for the species inhabiting wetland habitats; however, there are few examples of such application [44]. Therefore, our pre-filtration method may benefit the application of eDNA methods to turbid water, particularly wetlands although still remain several concerns particularly about contamination and time.

We suggest that pre-filtration is an effective method of preventing filter clogging and contributes to increased eDNA yield, high sensitivity, and high reproducibility; however this method may result in loss of DNA from the solution and an increase in costs. Several approaches for removing PCR inhibitors have been reported [45–47]. After eDNA extraction, eDNA re-purification by column [45, 48–50] can remove PCR inhibitors; however, this method results in low eDNA yield and has a high cost. In this study, we preliminarily evaluated the use of coffee filters for humic water and found that they could be useful for pre-filtering humic water. Fortunately, filtration can be conducted in parallel with pre-filtration, as such low-cost methods have also been found to reduce the inhibitory effect of humic water on PCR. Pre-filtration not only eases filtration and improves eDNA yields but also appears to reduce the levels of co-extracted PCR inhibitors, such as humic acids.

Here, we found that pre-filtration is a useful technique for removing PCR inhibitors, such as humic acid, in wetland habitats for both eDNA metabarcoding and species-specific detection by qPCR. Based on our results, we recommend using a mesh size of < 200 μm to obtain eDNA from the aquatic environment with lower PCR inhibition. This approach could also be applied in other habitats with turbid waters to optimise the filtration process.

## Supporting information

S1 Fig Boxplots showing the relationship between the number of detected species and the number of PCR replicates under each pre-filter condition. (TIF)

S2 Fig Reproducibility of fish communities by 12S rRNA amplicon analysis (MiFish) under each pre-filter condition. (A) Map showing the sampling points on 22 December 2018 as closed circles. This map was created using QGIS version 2.14 based on the map tile in the Geospatial Information Authority of Japan (https://maps.gsi.go.jp/development/ichiran.html). [Data source of the map tiles] Landsat8 image (GSI, TSIC, and GEO Grid/AIST), Landsat8 image (courtesy of the U.S. Geological Survey), and Submarine topography (GEBCO). (B) Venn diagram showing the number of shared species between each pre-filtration condition.
(TIF)

S3 Fig Two-dimensional NMDS ordination of the fish community under each pre-filtration condition based on Jaccard indices and Bray-Curtis indices for water samples collected on on June 9, 2018. Circles, squares, diamonds, triangles, and inverted triangles indicate the results for NoPre, Pre840μm, Pre200μm, Pre50μm, and Pre10μm, respectively. (A) Jaccard indices. NMDS stress was 0.21. (B) Bray-Curtis indices. NMDS stress was 0.032.
(TIF)

S4 Fig Two-dimensional NMDS ordination of the fish community under each pre-filtration condition based on Jaccard indices and Bray-Curtis indices for water samples collected on December 22, 2018. Circles, squares, diamonds, triangles, and inverted triangles indicate the results for NoPre, Pre840μm, Pre200μm, Pre50μm, and Pre10μm, respectively. (A) Jaccard indices. NMDS stress was 0.192. (B) Bray-Curtis indices. NMDS stress was 0.067.
(TIF)

S1 Table Taxonomic composition and sequence read numbers of the species detected in MiFish analysis under each pre-filtration condition. Water samples were collected from the Sagami River system on 9 June 2018, and 57 species were detected. The table shows the total read numbers obtained from eight PCR replicates of each sample.
(XLSX)

S2 Table Taxonomic composition and sequence read numbers of the species detected in MiFish analysis under each pre-filtration condition. Water samples were collected from the Sagami River system on 22 December 2018, and 69 species were detected. Compared to 9 June 2018, eight replicates of the first PCR were pooled before the second PCR.
(XLSX)

S3 Table Primer list used in the species-specific qPCR assay. CytB-F and CytB-R were used as primers for amplification. An EJ probe for TB was used as the detection probe for the THUNDERBIRD® qPCR Mix and an EJ probe for EMM was used as detection probe for the TaqMan Environmental Master Mix 2.0.
(XLSX)

S4 Table All threshold cycles of qPCR in this study. (XLSX)
