# Correction: Water pre-filtration methods to improve environmental DNA detection by real-time PCR and metabarcoding

**DOI:** 10.1371/journal.pone.0258073

**Published:** 2021-09-27

**Authors:** Kazuto Takasaki, Hiroki Aihara, Takanobu Imanaka, Takahiro Matsudaira, Keita Tsukahara, Atsuko Usui, Sora Osaki, Hideyuki Doi

Fig 5 is incorrect. The authors have provided a corrected version here.

**Fig 5:**
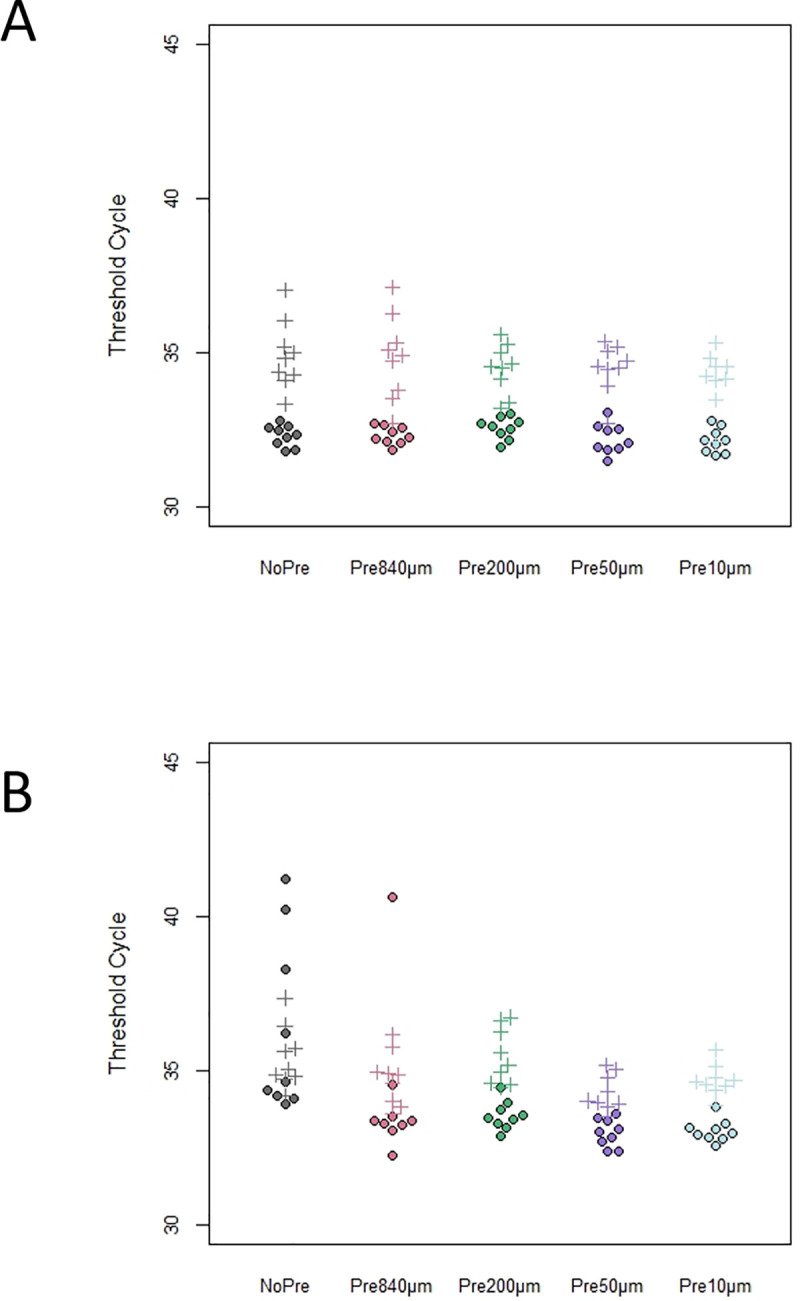
DNA detection assay (Threshold cycle, Ct) results for pale chub under each pre-filtration condition by qPCR. (A) In the absence of humin; B) in the presence of humin. Closed circles and daggers indicate the results detected by the THUNDERBIRD® qPCR Mix and Environmental Master Mix 2.0, respectively.
