# Supplementary figures and images for: Water pre-filtration methods to improve environmental DNA detection by real-time PCR and metabarcoding
Source: PLoS One. 2021 May 7;16(5):e0250162. doi: 10.1371/journal.pone.0250162 (PMC8104373; doi:10.1371/journal.pone.0250162)

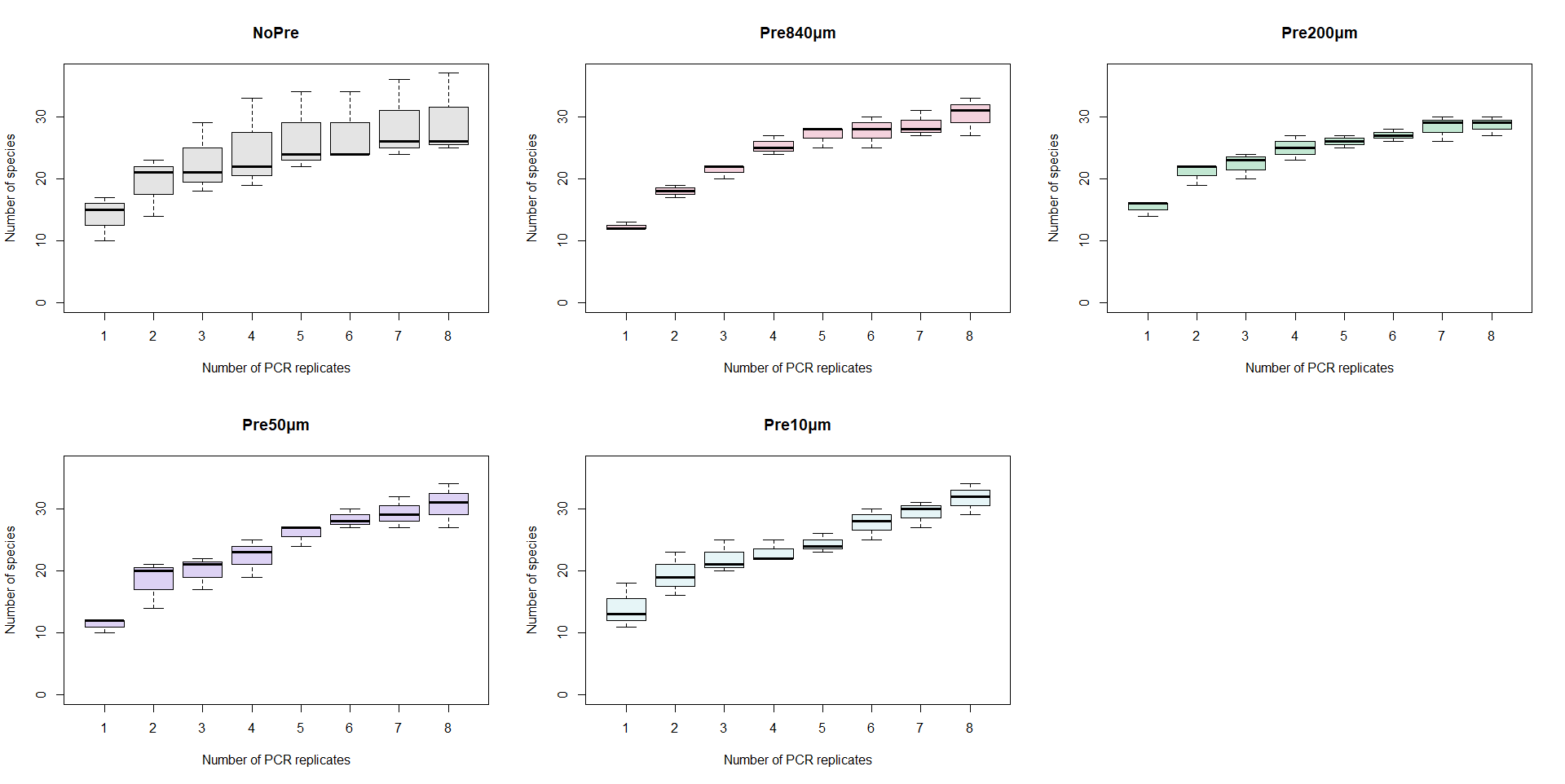

Supplement: S1 Fig — (TIF) [file pone.0250162.s001.tif]

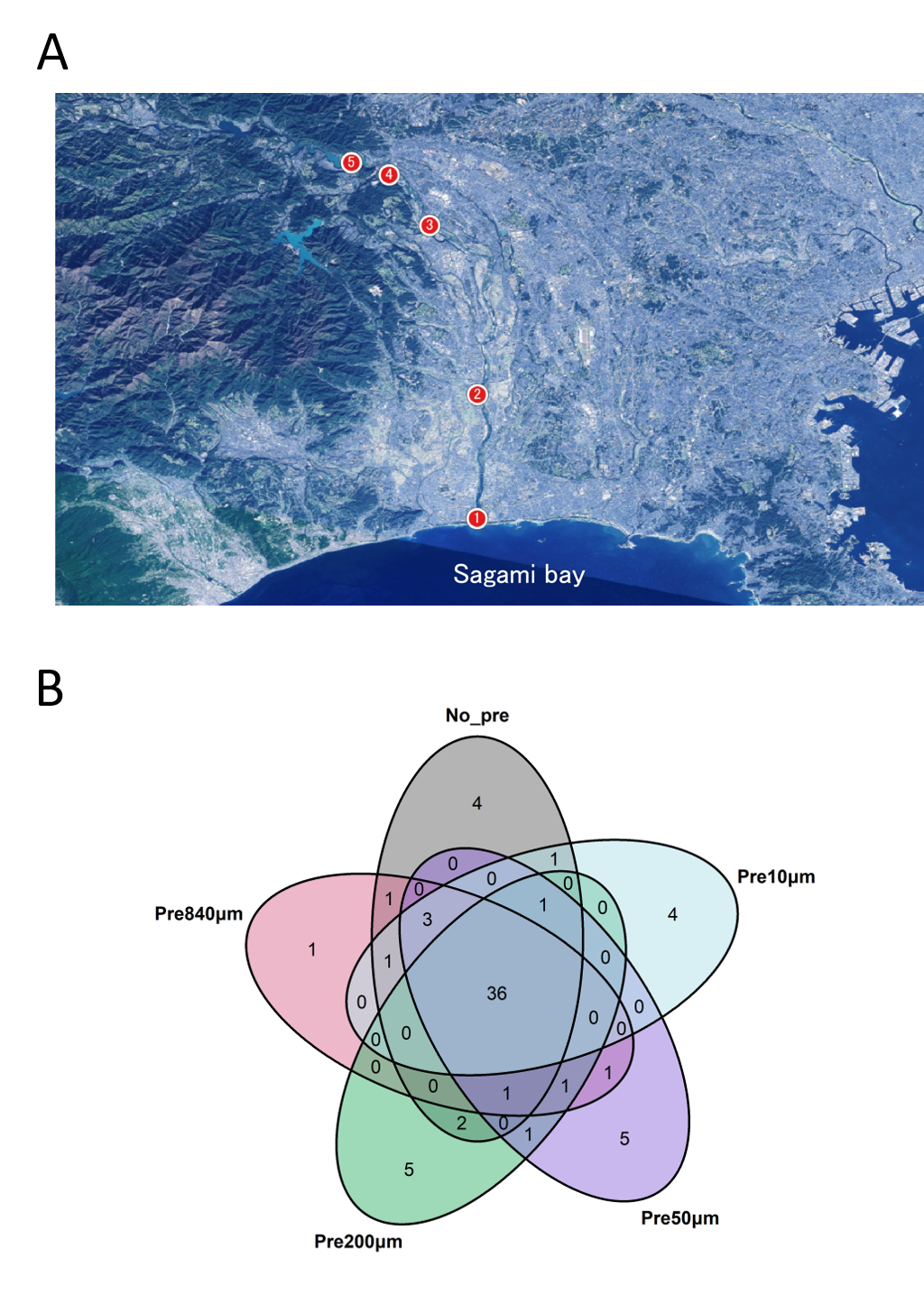

Supplement: S2 Fig — (A) Map showing the sampling points on 22 December 2018 as closed circles. This map was created using QGIS version 2.14 based on the map tile in the Geospatial Information Authority of Japan (https://maps.gsi.go.jp/development/ichiran.html). [Data source of the map tiles] Landsat8 image (GSI, TSIC, and GEO Grid/AIST), Landsat8 image (courtesy of the U.S. Geological Survey), and Submarine topography (GEBCO). (B) Venn diagram showing the number of shared species between each pre-filtration condition. (TIF) [file pone.0250162.s002.tif]

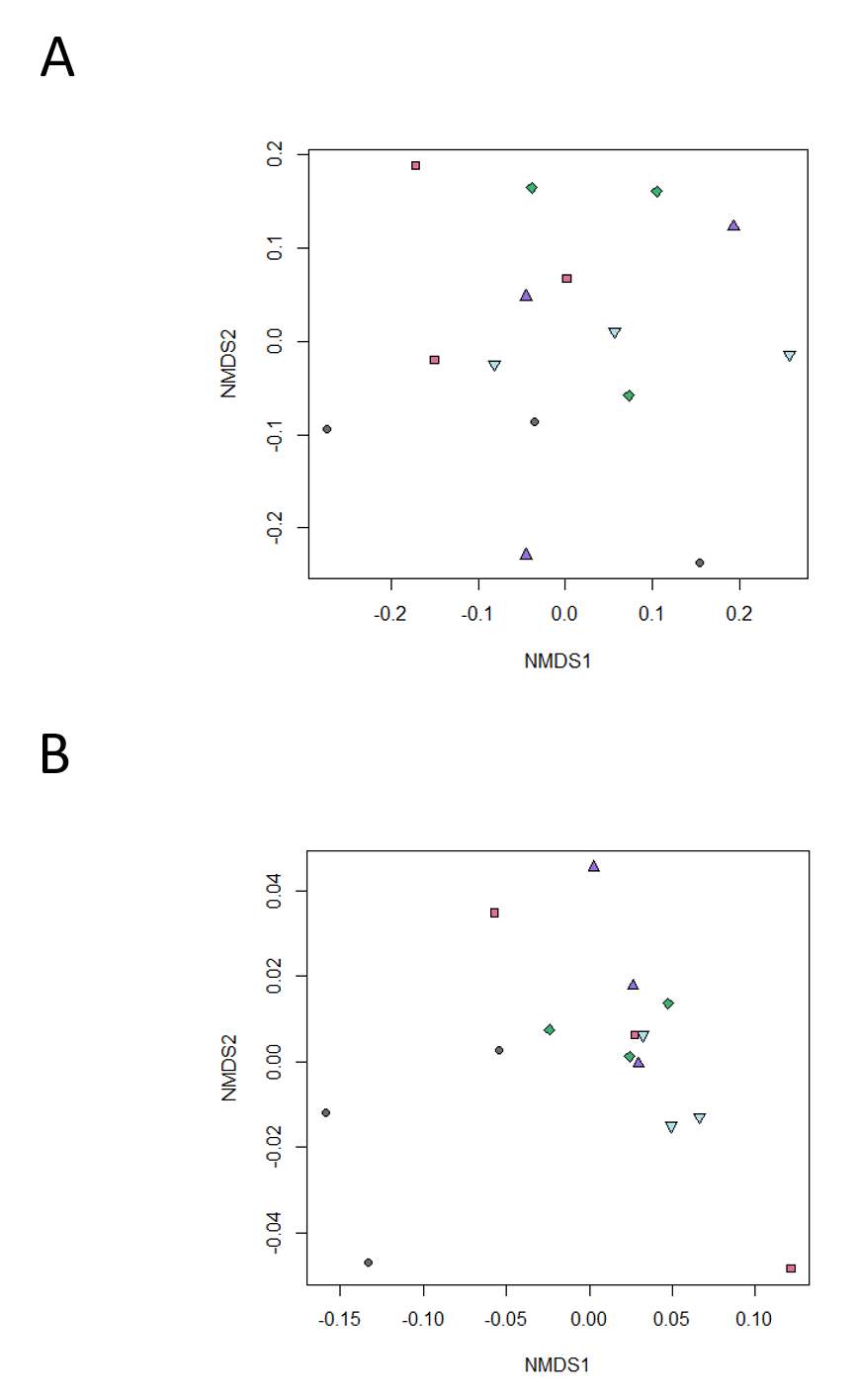

Supplement: S3 Fig — Circles, squares, diamonds, triangles, and inverted triangles indicate the results for NoPre, Pre840μm, Pre200μm, Pre50μm, and Pre10μm, respectively. (A) Jaccard indices. NMDS stress was 0.21. (B) Bray-Curtis indices. NMDS stress was 0.032. (TIF) [file pone.0250162.s003.tif]

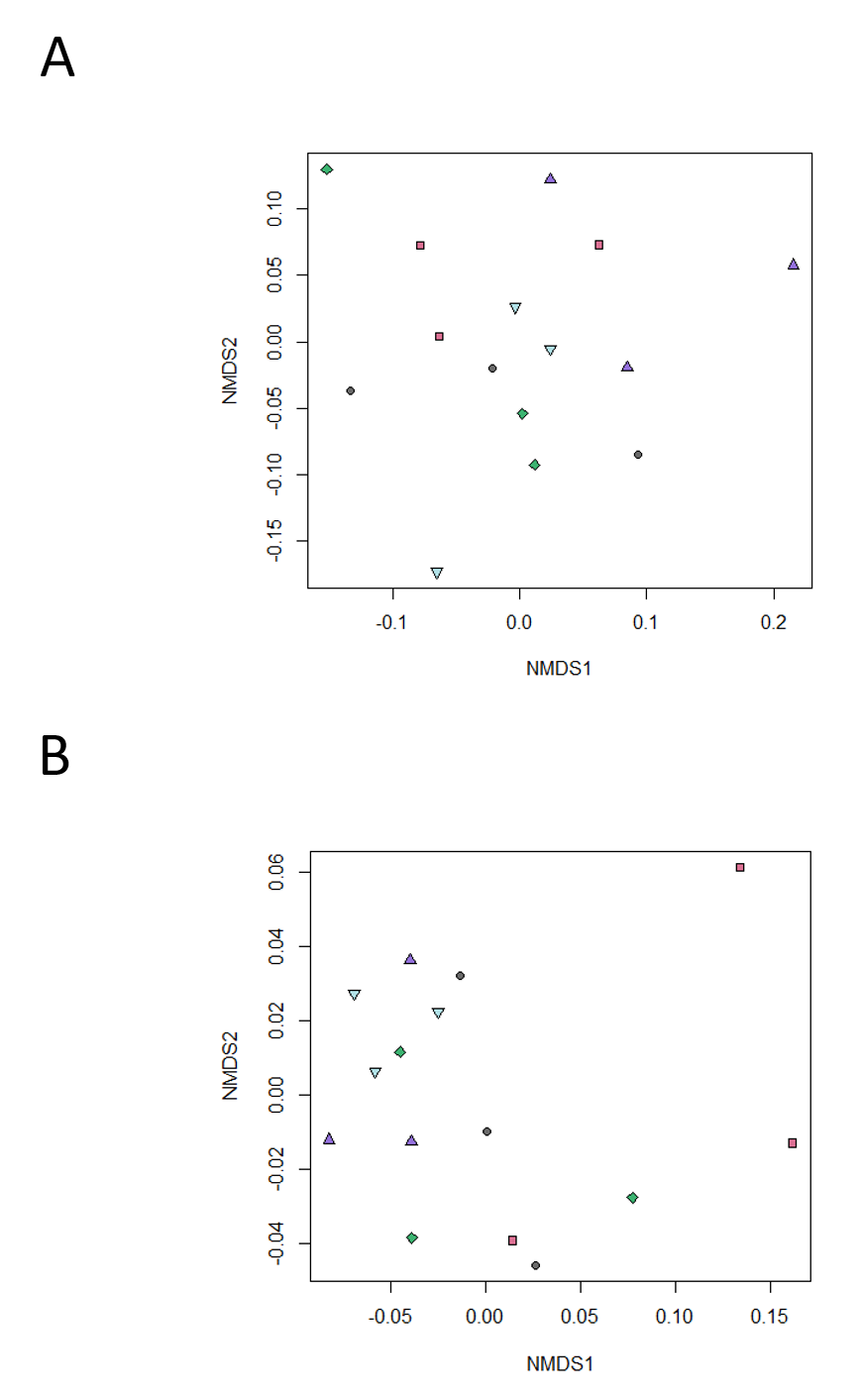

Supplement: S4 Fig — Circles, squares, diamonds, triangles, and inverted triangles indicate the results for NoPre, Pre840μm, Pre200μm, Pre50μm, and Pre10μm, respectively. (A) Jaccard indices. NMDS stress was 0.192. (B) Bray-Curtis indices. NMDS stress was 0.067. (TIF) [file pone.0250162.s004.tif]
